# Growing decision-making: the role of theory of mind, empathy, and personality traits in school-age children

**DOI:** 10.3389/fpsyg.2026.1708789

**Published:** 2026-02-03

**Authors:** Elisabetta Lombardi, Cinzia Di Dio, Ilaria Castelli, Davide Massaro, Antonella Marchetti, Annalisa Valle

**Affiliations:** 1 Department of Theoretical and Applied Sciences, eCampus University, Novedrate, Italy; 2 Department of Psychology, Research Unit on Theory of Mind, Research Centre on Theory of Mind and Social Competence in the Lifespan (CeRiToM), Università Cattolica del Sacro Cuore, Milan, Italy; 3 Department of Human and Social Sciences, University of Bergamo, Bergamo, Italy

**Keywords:** children decision making, empathy, personality traits, school-aged children, theory of mind

## Abstract

Children’s decision-making is a socio-cognitive skill embedded within a broader system that promotes understanding of others and effective management of interpersonal contexts, making it closely linked to Theory of Mind (ToM) and empathy. The present study examined how these abilities, together with personality traits and cognitive skills, relate to decision-making in middle childhood, specifically regarding fairness, altruism, and delay of gratification. A sample of 94 children aged 6–10 years completed tasks assessing fairness (Ultimatum Game), altruism (Dictator Game), and delay of gratification (Marshmallow Task), together with measures of ToM, empathy, personality traits, and cognitive ability. Results revealed that fairness was predicted by ToM and situational empathy, suggesting that acting fairly involves integrating mental-state reasoning with context-dependent emotional responsiveness. Altruism, in contrast, was specifically associated with affective empathy, indicating that children’s tendency to help others is primarily driven by their emotional sensitivity. Delay of gratification was unrelated to ToM or empathy. Instead, it was predicted by the personality trait of Openness to Experience, reflecting the role of trait-like motivational tendencies in delay of gratification. Age and general cognitive ability did not predict any of the decision-making outcomes. Path analyses confirmed that fairness and altruism are driven by social-cognitive mechanisms, whereas delay of gratification depends primarily on individual dispositions rather than interpersonal processing. These findings clarify the specific contributions of ToM, empathy, and personality to children’s decision-making and carry important implications for developmental and educational practices.

## 1 Introduction

Decision-making in childhood can be conceptualized as part of a broader social cognition system, encompassing the ability to understand others’ mental states, regulate emotions, and navigate social norms (Lombardi et al., 2017). Social decision-making arises at the intersection of cognitive and socio-emotional competencies, including Theory of Mind (ToM), empathy, and self-regulation. Middle childhood represents a critical developmental window, as key socio-cognitive abilities—such as mental-state reasoning, emotional understanding, and behavioral self-regulation—consolidate during this period (Carlson and Moses, 2001; Wellman, 2014). Moreover, important changes in fairness, altruism, and delay of gratification occur (Blake et al., 2014; Warneken and Tomasello, 2009), making this age range especially suitable for studying how children make prosocial decisions. Within this framework, children’s prosocial decisions—such as sharing or helping—reflect the development of both prosocial motivations and social-cognitive processing.

### 1.1 Decision-making in childhood

Research on children’s social decision-making often focuses on fairness and altruism (Lombardi et al., 2017, 2021), considered at the basis of prosocial behavior and moral judgment (Flook et al., 2019; Malti et al., 2016).

Fairness is conceptualized through inequity aversion, the tendency to resist inequitable situations. In social contexts, fairness guides people to forgo personal gain to re-establish equality. Fairness is assessed by the Ultimatum Game (UG), an economic game involving a Proposer and a Receiver, who share money (or goods). Fair receivers accept fair offers (balanced distribution) and reject unfair ones (disproportionate sharing). Children begin rejecting disadvantageous inequity around 3–4 years of age, and by 8, they also reject advantageous inequity, where they receive more than others (Smith et al., 2013).

Altruism refers to the tendency to help others achieve their goals and share valuable resources, with the aim of promoting both societal wellbeing and individual satisfaction. Children’s altruistic behavior emerges between 2 and 3 years (Warneken and Tomasello, 2013) and becomes stable during the school years (Benenson et al., 2007). Altruism, as a specific type of prosocial tendency/behavior, is assessed using the Dictator Game (DG), where the Proposer decides how much to offer the Receiver, who must accept whatever is given.

Another aspect closely linked to decision-making is delay of gratification, i.e., the ability to favor future, more substantial rewards over immediate ones, which is essential for pursuing long-term goals (e.g., success in academics; Casey et al., 2011). This ability is explored through the delay of gratification paradigm (Marchetti et al., 2014), which captures children’s capacity to resist immediate impulses in favor of more advantageous long-term choices. This ability emerges around 4 years and develops until 8–10 years of age, with the improvement of self-control and inhibition skills (Casey et al., 2011). Indeed, obtaining future rewards requires inhibiting immediate impulses. Whereas fair and altruistic decisions offer immediate benefits to others and help foster long-term positive outcomes for the community, choosing to delay gratification generally has benefits that are more centered on the individual (Casey et al., 2011). Although delaying gratification can, in broader real-life contexts, have interpersonal or socially relevant consequences, in the classic Marshmallow Task, the benefits are strictly individual: the child decides whether to obtain a larger personal reward for themselves by waiting.

This evidence suggests that different psychological characteristics underlie these decisions. Social abilities, such as ToM and empathy, may relate more to fair and altruistic decisions, while individual traits, such as cognitive skills, may influence delaying gratification.

### 1.2 Theory of mind and empathy

Effective social interactions require understanding not only people’s observable actions but also the underlying mental and emotional states that drive them. Two main competencies involved in this process are ToM and empathy. ToM, or mentalization, is the capacity to attribute mental states (beliefs, desires, intentions, etc.) to oneself and others to predict behaviors and is classically assessed through recursive-thinking reasoning of increasing complexity (Astington et al., 2002). By age four, children can represent others’ minds, and by late adolescence, they can engage in third-order recursive reasoning, for example, “I think that you think that he thinks that another thinks x” (Valle et al., 2015).

Empathy is considered a multifaceted construct, consisting of both a cognitive and an emotional dimension. In infancy, empathy begins as emotional contagion, namely “emotional empathy,” involving automatic, simulation-based sharing of others’ emotions; during childhood, empathy develops into “cognitive empathy,” involving deliberate perspective-taking to understand others’ emotional states (Shamay-Tsoory et al., 2009). Moreover, empathy can be conceptualized either as “general affective empathy,” a dispositional tendency to respond to others’ emotions, or as “situational (state) empathy,” an emotional reaction triggered by a specific context (Baldner and McGinley, 2014).

Schurz et al. (2021) consider ToM and empathy “umbrella terms” indicating different social cognitive processes with limited convergence. From a neurocognitive perspective, the two constructs represent a continuum of related processes, as reflected in their partially overlapping neural regions (Cerniglia et al., 2019). Accordingly, they can be viewed as functionally distinct yet neurocognitively intertwined systems that play crucial roles in prosocial behavior (Imuta et al., 2016).

### 1.3 Social cognition and decision-making in children

Regarding fairness-related decisions, inequity aversion gradually shifts from an early egocentric focus on self-gain toward more norm-based and reciprocal thinking as ToM develops. Studies using the UG indicate a correlation between ToM and fairness (Steinmann et al., 2014): older children with more advanced ToM—particularly second-order ToM—take the partner’s perspective into account and increasingly reject unfair offers (Castelli et al., 2017; Lombardi et al., 2017). However, the role of empathy in children’s fairness decisions remains less clear. Tsoi and McAuliffe (2019) reported that children’s ToM predicted inequity aversion, whereas empathy (parent-reported) did not.

The relationship between ToM, empathy, and the prosocial tendency toward altruism is still debated. Although empathy supports early-emerging prosocial motivations, evidence for a direct link with altruism is mixed. In contrast to the classical perspective, Rose et al. (2024) found no association between empathy and altruism in middle childhood and showed that higher levels of ToM appear to make children more strategic in their social decisions, but also less altruistic. Other studies show that ToM predicts sharing in children (Takagishi et al., 2010; Wu and Su, 2014), while Liu et al. (2016) found that neither ToM nor inhibitory control predicted pure altruistic sharing in a DG with anonymous partners. These discrepancies likely reflect differences in task structure (strategic vs. pure altruism) and measurement of ToM (false belief vs. advanced mentalizing).

Studies on delay of gratification, another core component of children’s decision-making, show that early first-order ToM supports the ability to choose larger delayed rewards over immediate ones (Marchetti et al., 2014). In adults, higher ToM is also associated with delaying gratification (Launay et al., 2015). To the best of our knowledge, no studies have examined whether empathy and ToM jointly contribute to delaying gratification, leaving an open question about whether prosocial and self-regulatory decisions rely on shared or distinct socio-cognitive processes.

### 1.4 Personality traits, cognitive abilities, and decision-making

We also considered personality traits as an essential component of individual differences relevant to children’s decision-making. Given the central role of personality in predicting behavior, even in early development (Wright and Jackson, 2022), their inclusion allows for a more comprehensive and multidimensional understanding of the factors shaping fairness, altruism, and delay of gratification. Among Big Five traits, Openness is theoretically linked to perspective-taking, curiosity about others’ mental states, and sensitivity to social norms. Recent meta-analytic evidence shows that Openness is positively associated with prosociality in economic games (Thielmann et al., 2020), supporting its inclusion as a relevant individual difference in children’s social decision-making. This choice is grounded in two main considerations. First, previous studies have documented associations between Big Five traits and prosocial tendencies in childhood (Allgaier et al., 2020); second, the contribution of personality to delay of gratification remains largely underexplored. In this regard, we focused especially on traits linked to cognitive flexibility—such as Openness to Experience—which may facilitate the inhibitory control, self-regulation, and self-distraction strategies required to resist an immediate reward in favor of a delayed, larger benefit.

Cognitive abilities, particularly fluid reasoning, support effective decision-making by enabling the evaluation of complex information and the use of goal-directed strategies. Individuals with higher levels of intelligence are more likely to engage in model-based rather than habitual strategies (Maran et al., 2020) and to display more adaptive and coherent strategic behavior in decision-making (Proto et al., 2019); moreover, fairness and altruism are affected by numerical cognition and cognitive flexibility, the latter moderating the relationship between ToM and fairness (Wang et al., 2023).

By integrating ToM, empathy, personality, and cognitive skills, the present study aims to provide a perspective on how children’s decision-making emerges from the interplay between social cognitive and personality dimensions.

## 2 Aims

This study aimed to investigate the relationship between decision-making, ToM, empathy, personality traits, and cognitive abilities in school-aged children, focusing on:

- How ToM and empathy (both affective general and situational empathy) relate to fairness (assessed through the UG) and altruism (assessed through the DG).
- The role of personality traits and cognitive abilities in the delay of gratification performance.
- Test an integrated structural model capturing the joint pathways linking social cognition, personality, and decision-making in middle childhood.

We expected ToM and empathy to be more strongly associated with fairness and altruistic behavior (UG and DG), which rely on social reasoning, whereas cognitive ability and personality traits are expected to be more strongly related to delay of gratification (Marshmallow Task).

## 3 Materials and methods

### 3.1 Participants

Ninety-four (94) children (42 girls) between 6 and 10 years old (M = 102 months, SD = 19 months) were recruited from two public primary schools in the North (*N* = 43) and Centre (*N* = 51) of Italy. The sample size was determined by the number of eligible children available in the participating schools during the data collection period. No *a priori* power analysis was conducted. However, a *post hoc* sensitivity analysis using GPower for multiple regression (*α* = 0.05, power = 0.80, 6 predictors) showed that with *N* = 94, the study has adequate power to detect at least medium effect sizes (f^2^ = 0.15). For the analysis, inclusion criteria were fluency in Italian and absence of developmental or learning disorders. Parental informed consent was obtained from each participant. The Scientific and Ethics Committee of the Department of Psychology of the Catholic University of Milan (Italy) approved the research, following the Helsinki Declaration.

### 3.2 Measures

Socio-economic status (SES) was measured by the Family Affluence Scale (FAS; Currie et al., 2008), a 4-item questionnaire on family wealth completed by children. The total score, ranging from 0 (lowest affluence) to 9 (highest affluence), serves as an index of socio-economic background.

Fairness and Altruism were assessed using the Ultimatum Game (UG; Güth et al., 1982) and the Dictator Game (DG; Kahneman et al., 1986), played for real. At the beginning of each individual session, the experimenter gifted each child 10 collectible cartoon-themed cards—chosen by the child from two types offered. These cards were rewarding in and of themselves: they could not be exchanged for other goods and were kept by the children at the end of the session. In both games, children acted as Proposers and allocated their cards to a Receiver represented by a neutral, gender-matched child drawing (identical for all participants; see Castelli et al., 2010; Lombardi et al., 2017). Before each game, the experimenter told the Proposer that he/she would be playing with an anonymous child from a different class, represented by the drawing. In the UG, the Proposer was told that the Receiver could accept or reject the offer: if accepted, the cards would be divided as proposed; if rejected, neither child would receive any cards. After the Proposer made the offer, the experimenter left the room, stating that she was going to show the offer to the Receiver and, upon returning, reported that the offer had been accepted (everyone’s proposals were systematically accepted, without the child’s knowledge). In the DG, the Proposer was informed that the Receiver could not refuse the allocation; the experimenter told the child that the offer would be delivered to the other child at the end of the experimental session. One round per game was administered, and the amount offered was recorded.

Delay of Gratification was assessed by the Marshmallow Task (MT; Mischel et al., 1972), where the child could take two cards immediately or wait for the researcher’s return (15 min) to gain four cards. The score is the number of seconds the child waits.

*Theory of Mind* was assessed by three second-order false-belief tasks (FBT; Astington et al., 2002); Look-prediction Task and Say Prediction Task (Wimmer and Perner, 1983), and the Ice-Cream Man Task (Perner and Wimmer, 1985). Each task consists of a story accompanied by a picture; subjects are asked to reason about what one person believes another person believes. Tasks involving the unexpected transfer of an object by a character, witnessed by the hidden protagonist, were used. The test question asks where the character thinks the protagonist will go to look for the moved object. To answer correctly, the subject must put themselves in the shoes of the character who has a false belief about the protagonist’s mental representation of reality. The questionnaire is composed of two control questions, a first-order question, a second-order question, and a justification question. Each correct answer is awarded one point, for a total of 5 points for each task (total score = 15).

*Empathy* was assessed by two tasks, the Index of Empathy (IE; Bryant, 1982) and How I Feel in Different Situations (HIFDS; Feshbach et al., 1991; Bonino et al., 1998). The IE is a questionnaire with 22 statements (*α* = 0.80; total score = 0–22) that assesses dispositional, general affective empathy—namely stable tendencies to experience sadness or concern for others (de Wied et al., 2007). HIFDS is a self-report questionnaire with 12 items that measure situational or state empathy (Baldner and McGinley, 2014), capturing children’s immediate affective and cognitive reactions to specific scenarios. HIFDS is rated on a 4-point scale; each scale—both the affective and the cognitive one—has a total score of 0–24, and the scale scores were summed to obtain a total score (α = 0.76).

*Personality Traits* were assessed using the Big Five Questionnaire – Child (BFQ-C; Barbaranelli et al., 1998), comprising 65 items rated on a 3-point Likert scale (score range 13–39), measuring Extraversion, Neuroticism, Openness, Agreeableness, and Conscientiousness (α range = 0.839–0.892).

*Cognitive abilities* were assessed by Raven’s Colored Progressive Matrices (CPM; Raven, 1984), a paper-and-pencil task with 36 increasingly difficult items. Each correct option scores 1 point (total score = 0–36).

### 3.3 Procedure

Children were tested during one collective session and two individual sessions, conducted in the same week. All tasks were administered in a paper-and-pencil format by the same experimenter, in a quiet room of the school; no other adult was attending. In the collective session, the experimenter divided each class in groups of 5–7 children and administered the FAS, the BFQ-C, the HIFDS and the IE (about 30 min); in the first individual session, in a quiet room in the school, the experimenter administered the CPM, the MT, and DG (about 20 min); in the second individual session, the experimenter administered the FBT, and the UG (about 20 min). In each session, the order of the tasks was randomized.

### 3.4 Data analysis

Descriptive statistics and Pearson’s correlations were first computed to provide an overview of the bivariate relations among all variables. To show the unique contribution of ToM, empathy, personality traits, and cognitive abilities to decision-making, we conducted three separate multiple regression analyses—one for each outcome (UG, DG, MT)—using the same theoretically grounded set of predictors (Heinze et al., 2018): ToM, general affective empathy, situational empathy, the five personality traits, cognitive ability controlling for the children’s age, gender, SES as suggested by literature (Wang et al., 2023).

Finally, the path analysis (SEM) using the jAMM package in Jamovi (Version 2.3.21) was conducted as a descriptive complement to the regression analyses to illustrate the joint pattern of associations among social-cognitive, personality, and decision-making variables.

## 4 Results

**Figure 1:**
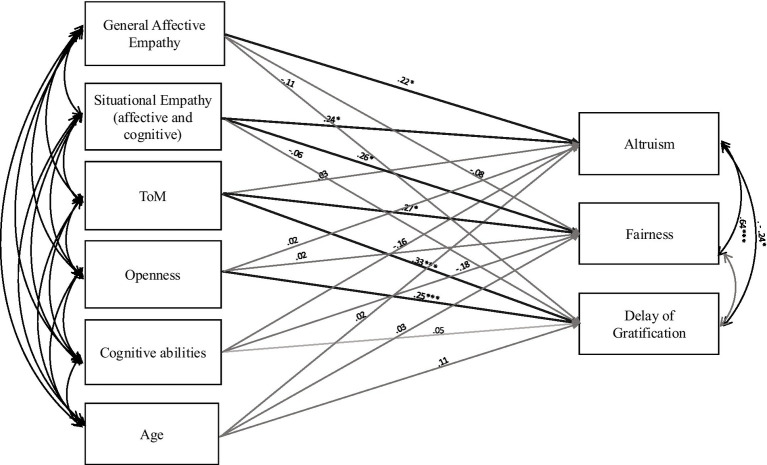
Path diagram. Rectangle represents observable variables. Single-beaded arrow represents the regression path, the arrow in bold represent only the significant regression path. Double-headed arrows represent the correlations between the variables. All the coefficients are standardized.

As shown in Table 1, Pearson correlation analyses reveal associations between decision-making tasks (DG, UG, MT), Social cognition tasks (FBT, IE, HIFDS), and personality traits (BFQ-C; Agreeableness, Emotional instability, Openness to experience, Extraversion, Conscientiousness), beyond the socio-demographic variables (SES, age) and cognitive abilities (CPM). DG and UG performance correlate with social cognition, but do not exhibit significant associations with age, CPM, and personality traits. More specifically, UG score is positively correlated with both general affective empathy and situational empathy, and ToM (and with DG score), and DG score is positively correlated with both empathy tasks assessed. MT is strongly related to age, CPM, Openness to experience, and FBT. As expected, several predictors showed significant zero-order correlations with decision-making outcomes. However, when entered simultaneously into the regression models, some associations weakened or became non-significant, reflecting shared variance among theoretically related predictors (e.g., ToM and empathy). Thus, regression coefficients estimate each predictor’s unique association with the outcome while controlling for overlap with other variables in the model. The regression model predicting fairness (UG) was significant, F_(11, 82)_ = 2.47, *p* = 0.010, explaining 25% of the variance (R^2^ = 0.25, R^2^__adjusted_ = 0.15). Within the unified predictor set, situational empathy (*β* = 0.31, *p* = 0.018) and ToM (β = 0.24, *p* = 0.031) were significant predictors of fairness, whereas age, gender, SES, personality traits, general affective empathy, and cognitive ability were not. The model predicting altruism (DG) was also significant, F_(11, 82)_ = 2.60, *p* = 0.007, accounting for 26% of the variance (R^2^ = 0.26, R^2^__adjusted_ = 0.16). In this model, general affective empathy (β = 0.25, *p* = 0.043) and situational empathy (β = 0.30, *p* = 0.020) significantly predicted altruism, whereas ToM, personality traits, cognitive ability, and demographic variables were not significant predictors. Finally, the delay of gratification (MT) was significant, F_(11, 82)_ = 3.55, *p* < 0.001, explaining 32% of the variance (R^2^ = 0.32, R^2^__adjusted_ = 0.23), with Openness to Experience (β = 0.23, *p* = 0.038) and ToM (β = 0.35, *p* = 0.003) as the only significant predictors. Path-analysis (Figure 1) examined the structural pathways linking empathy, Theory of Mind, personality, and cognitive abilities to children’s decision-making. The model showed a good fit to the data, as indicated by a non-significant chi-square (X^2^ = 4.08, df = 4, *p* = 0.371). Additional fit indices further supported model adequacy, with low SRMR (0.027), and RMSEA (0.014, 95% CI [0.000, 0.141], *p* = 0.51), and excellent comparative fit indices (CFI = 0.998; TLI = 0.989).

**Table 1:** Correlations between decision-making tasks and all the variables.

Variable	M	SD	AGE	FAS	CPM	BFQC-C	BFQC-O	BFQC-A	BFQC-E	BFQC-EI	HIFDS	IE	FB	MT	DG
1. FAS	6.17	1.76	0.174	—											
2. CPM	28.6	4.91	0.700***	0.147	—										
3. BFQC—conscientousness	29.1	4.63	−0.024	0.112	−0.022	—									
4. BFQC—openness	30.2	4.42	−0.004	0.234*	0.060	0.695***	—								
5. BFQC—agreeableness	31.7	4.34	−0.022	0.172	0.048	0.529***	0.524***	—							
6. BFQC—extraversion	34.5	4.07	0.075	0.212*	−0.113	0.466***	0.465***	0.486***	—						
7. BFQC—emot. Instability	19.6	4.04	0.030	0.030	−0.011	0.003	−0.138	−0.116	0.065	—					
8. HIFDS—empathy	28.1	7.27	0.039	0.260*	0.090	0.232*	0.305**	0.398***	0.300**	0.030	—				
9. IE—empathy	13.7	3.40	0.139	0.164	0.144	0.179	0.202	0.366***	0.143	−0.001	0.555***	—			
10. FB—TOM	11	2.41	0.450***	0.060	0.425***	−0.038	0.132	−0.077	−0.023	−0.184	0.236*	0.211*	—		
11. MT—delay of gratification	739	263	0.305**	0.187	0.287**	0.189	0.299**	0.069	0.061	−0.199	0.059	0.018	0.438***	—	
12. DG—altruism	3.70	2.06	−0.006	0.103	−0.058	−0.047	0.068	0.047	−0.028	0.131	0.370***	0.358***	0.084	−0.173	—
13. UG—fairness	4.07	1.66	0.064	−0.023	0.001	0.030	0.174	0.127	0.036	−0.063	0.369***	0.281**	0.291**	−0.007	0.677***

* *p* < 0.05, ** *p* < 0.01, *** *p* < 0.001.

## 5 Discussion

The present study examined how children’s decision-making is shaped by distinct yet interconnected components of social cognition, such as ToM and empathy, personality, and cognitive abilities. By investigating fairness, altruism, and delay of gratification, our findings provide a comprehensive picture of the socio-cognitive and trait-based mechanisms that underlie different forms of decision-making in middle childhood.

Our findings suggest that both ToM and empathy predict fairness. While the association between ToM and fair decisions aligns with existing literature (Marchetti et al., 2014), the role of empathy extends previous evidence from adult samples (Bock and Goode, 2007). Notably, situational, rather than general affective empathy, influences fair decisions. Situational empathy integrates affective and cognitive dimensions of empathy and reflects children’s ability to understand the other’s mental states and apply strategic prospective thinking in specific situations. These results suggest that empathic abilities, when strategically and contextually applied, are crucial for acting fairly in social interactions. What matters is not general empathic activation, but the ability to regulate and apply empathy in context to make equitable choices.

Our results further evidence that altruistic decisions are associated with both general affective empathy and situational empathy (as indicated in the studies regarding the link between empathy and prosocial behavior; Kozloff et al., 2021), rather than with ToM. This pattern is consistent with a large body of research showing that affective empathy strongly predicts children’s prosocial behavior. At the same time, our findings highlight that situational empathy also plays a role, suggesting that children rely not only on their general empathic disposition but also on their strategic context-specific emotional responsiveness when making altruistic decisions. Although this form of situational empathy includes a cognitive component, ToM itself does not appear to contribute to altruistic choices: pure perspective-taking is not recruited in altruism, indicating that acting altruistically requires sharing the other’s emotional state rather than merely understanding it from a cognitive standpoint.

The capacity to delay gratification is related to both ToM abilities and the personality trait of Openness to Experience. The former aligns with research in childhood showing that ToM depends on executive skills that also support children’s delay gratification (Carlson and Moses, 2001; Carlson et al., 2004; Marchetti et al., 2014). The association with Openness to Experience—defined as a preference for novelty and exploration—suggests that personality may contribute to early individual differences in delay of gratification. Although much of the research linking personality to decision-making has been conducted with adult samples—for example, studies documenting associations between Openness to Experience, Neuroticism, and prosocial or donation behaviors (Pinazo et al., 2016; Thielmann et al., 2020)—our findings suggest that these patterns may begin to emerge as early as middle childhood.

We propose that an inclination toward new experiences may require inhibiting immediate, habitual responses to familiar options, allowing children to take the time needed to evaluate how best to engage with novelty. For those high in Openness to Experience, the reward value of novelty may outweigh the comfort of routine, thereby supporting their ability to delay gratification. Taken together, these findings suggest that the Openness to Experience may play an emerging role in shaping children’s decisions to delay gratification.

Finally, neither cognitive ability nor age directly predicted decision-making outcomes in our model. However, this null effect should be interpreted with caution. Although these variables did not explain variance beyond that accounted for by ToM and Openness to Experience, both were correlated with these predictors and delay of gratification. This pattern suggests that age and cognitive ability may contribute indirectly to children’s decisions, through socio-cognitive or personality-related mechanisms.

This interpretation is consistent with recent findings showing that specific executive function components—such as attention and inhibitory control—are more relevant predictors of altruism than general cognitive ability (Rose et al., 2024). In our study, cognitive ability was assessed as a broad construct rather than through targeted executive-function measures, which may explain the absence of direct effects. Similarly, children in middle childhood may already possess sufficient skills to perform the tasks, while age-related differences may still operate indirectly through developmental changes.

## 6 Conclusion

Overall, these findings offer insights into the complex interplay between social cognition and personality traits in developing fairness, altruism, and delayed gratification, emphasizing the importance of social cognitive skills and personality factors, rather than age or general cognitive ability, in guiding prosocial decisions during childhood.

However, some limitations should be acknowledged. Although the sample size was adequate for the analyses, it limits the generalizability of the findings, and the cross-sectional design precludes causal inferences. Unmeasured factors, such as specific executive functions beyond global cognitive ability, may partly account for the observed associations; future studies could examine whether affective empathy moderates the association between ToM and prosocial tendencies. Longitudinal designs and more fine-grained measures of executive functions would help clarify the developmental mechanisms underlying decision-making and delay of gratification, as well as the stability of fairness and altruism across different social contexts (e.g., peers vs. unfamiliar partners). From an educational perspective, interventions aimed at fostering empathy, perspective-taking, and Openness to Experience may help promote prosocial decision-making and self-regulation in children.

## Data Availability

The raw data supporting the conclusions of this article will be made available by the authors, without undue reservation.
